# On illness and value: biopolitics, psychosomatics, participating bodies

**DOI:** 10.1136/medhum-2018-011588

**Published:** 2019-06-07

**Authors:** Monica Greco

**Affiliations:** Department of Sociology, Goldsmiths, University of London, London SE14 6NW, United Kingdom

**Keywords:** philosophy of medicine and healthcare, psychosomatic medicine, social sciences, politics, psychoanalysis

## Abstract

In its heyday, around the mid-twentieth century, psychosomatic medicine was promoted as heralding a new science of body/mind relations that held the promise of transforming medicine as a whole. Sixty years on, the field appears to have achieved no more than a respectable position as a research specialism within the medical status quo. This paper articulates the problematic of psychosomatics through a number of propositions that reconnect its promise of novelty to the present and to contemporary concerns. In contrast to classic approaches to ‘psychosomatic problems’, which typically set out by denouncing the conceptual inadequacy of mind/body dualism, the focus proposed is on the resilience of dualism as an empirical datum deserving closer analysis. The paper thus asks: what is the character of dualism considered under the aspect of what it achieves, and thus as an expression of value? Drawing on the thought of A N Whitehead, Michel Foucault and Viktor von Weizsäcker, the argument formulates a set of ‘psychosomatic problems’ informed by the concept of biopolitics and introduces their relevance in relation to the politics of participatory medicine.

There are stories that need to be ceaselessly reactivated in order to be relayed with new givens and new unknowns.1

In 1956, to mark the centenary of Freud’s birthday, Felix Deutsch wrote a short essay in Freud’s memory that focused on the relationship between medicine (‘the mother science’) and psychoanalysis (‘her most brilliant child’). The essay was entitled ‘The Riddle of the Mind-Body Correlations’ and Deutsch had prepared it for a workshop on ‘psychosomatic problems’ as the starting point for a group discussion. The group would meet regularly for the subsequent 3 years under the aegis of the Boston Psychoanalytic Society, and their conversations would crystallise into a series of papers published as an edited book, with the title: *On the Mysterious Leap from the Mind to the Body – A study on the Theory of Conversion* (1959).

This paper aims to inaugurate a new set of conversations on the problematic of psychosomatics, by reclaiming the relevance of some propositions originally formulated in that field. My first step in this endeavour is to reactivate the story of the discussions convened by Deutsch—a story that, together with the question of the medical value of psychoanalysis, has since been largely relegated to obsolescence. To reactivate the story means to retrieve what it may be still capable of conveying as a lure for contemporary thought, in the context of ‘new givens and of new unknowns’.^1^ Taking the discussions as a starting point, my argument will proceed by conjugating *psychosomatics* with the concepts of *biopolitics* and of *participation*, to produce a reframing of the problematic of psychosomatics for contemporary times.

## Mysteries, riddles, and the resilience of dualism

The phrase *mysterious leap from the mind to the body* had been used by Freud in 1917, and Deutsch was already proposing a ’reactivation’ when he chose it as the theme for his workshop.2 In preparation for the event, participants were asked to offer preliminary opinions on a specific question: was the reference to a ‘mysterious leap’ still warranted, or had ‘the known psychophysiological facts’ by then ‘clarified the “mystery” that had puzzled Freud’?3 The ensuing discussion now comprises chapter 2 of the book Deutsch eventually published. This chapter—where the name of each participant is followed by their response to the question, some ranging over several pages, others barely a paragraph long—vividly preserves for the reader the sense of their mutual presence, as if the text were repeating the gesture of eliciting contributions by going around the room. Similarly, it preserves a sense of incommensurability between the different responses, without attempting to elide them into a single coherent account. From this it is apparent that participants interpreted the task posed by the initial question in very different ways. On the one hand, some clearly understood it as a philosophical invitation to express their views on the ‘mind/body problem’. In this vein, they tended to respond by endorsing versions of what they called a ‘monistic approach’ or ‘monistic concept’, to argue that ‘there is no separation between psyche and soma’ and therefore also ‘no leap, no mystery’.4 In support of this view they cited the difference and integration between levels of organic complexity, using various examples; in some cases they also pointed to the ideas of process-oriented philosophers like William James, Henri Bergson and Alfred North Whitehead, among others. By contrast, several members of the group referred the question to something quite different, namely to the clinical and pragmatic task of accounting for the relation between ‘mind’ and ‘body’ as ‘fantasy conceptions’ underpinned by affective investments. In the words of Peter H Knapp:

All patients have vague, empirical, naïve fantasy conceptions of something they call their body and something they call their mind. Phenomena that appear to originate in one area seem to lead to effects in the other. These … conceptions are important in the thinking of individuals and are useful in understanding many of the clinical data which they present.5

From this clinical perspective, accounting for the ‘leap’ from the psychic to the physical meant investigating the ontogenetic origins of the differentiation between experiences of ‘mind’ and ‘body’, as well as the implications of that differentiation for homoeostatic regulation and for clinical presentations, using the techniques and concepts of psychoanalysis. Conceived in this way, the question Deutsch had posed to the group appeared in fact to be ‘less a question of a leap than that of a gap, that is, in our knowledge … which has to be filled’.6

Despite these significant differences, all participants appeared to share in the optimistic vision of a future where, through the cumulative advance of observations backed by a philosophical consensus over monism, mind/body relations would come to be clearly articulated in the context of a new science called ‘psychosomatic medicine’. In this sense, Freud’s *mystery* had become Deutsch’s *riddle*: still a difficult problem but one that, unlike mysteries, held the promise of a solution; moreover a solution that would transform medicine as a whole. This ambitious expectation had been shared by leading figures in psychosomatic medicine since the early days of the movement in the 1920s and 1930s, only to be incrementally disappointed and revised in subsequent decades.7

As we revisit this story 60 years later with a view to articulating what the notion of ‘psychosomatic problems’ might signify today, the first new given for us to think with is the historical failure of the ambition of psychosomatics—that is, the failure of psychosomatic medicine to have achieved more than a respectable, yet marginal position as a specialist field of research within the biomedical status quo. As we acknowledge this background, however, we must also acknowledge that the failure itself constitutes something more than a mere negativity: it testifies, rather, to the *resilience* of the dualism that Deutsch and his associates had tended to dismiss, whether as a philosophical mistake, a naïve vernacular ‘fantasy’ or a surface impression reflecting the provisional lack of an adequate method of scientific observation. This resilience appears all the more remarkable in light of the fact that, in the years since the workshop and the publication of the book, evidence of ‘mind-body correlations’ has indeed accumulated steadily- enough to warrant the emergence of new transdisciplines like psychoneuroendocrinoimmunology, and the continued existence of the journal *Psychosomatic Medicine* (among others). To the extent that these have a claim to heralding a fundamental transformation of the dominant medical model, however, this claim remains largely unheard or ignored by the mainstream.8 Rather, the contrary seems to have happened: the ambition of psychosomatic medicine has adapted to constraints implicit in the dominant model. In his study of editorial processes and decisions at *Psychosomatic Medicine*, for example, Nassim Mitzrachi has documented a fundamental shift ‘from causation to correlation’ in the focus of the journal over the four decades from 1939 (year of its foundation) to 1979.9 This shift, he claims, occurred as a result of a complex interplay between internal and external forces in shaping the editors’ efforts to achieve legitimacy for the field in the wider medical community. While the scope of Mitzrachi’s explanation is limited, he is right in concluding that the correlational models that gradually came to replace the search for causal mechanisms ‘presupposed and reduplicated the split the [journal’s] founders ironically sought to supersede’.10 Instead of spearheading a revolution in medical thought and practice, the impulse of psychosomatic medicine has therefore itself been reabsorbed and reconverted into the dualist mainstream.

The *resilience of dualism*, then, rather than the supposed fundamental unity of mind and body, is what I propose we take as the point of departure for a discussion of the problematic of psychosomatics today. In other words, I propose we do not start by considering dualism as a philosophical problem or even as a scientific problem, a riddle whose solution would rest on the development of an adequate method of investigation. Rather, I propose that we approach modern dualism in the first instance as an *empirical and historical datum*, a given. And that we formulate our questions not in abstraction from this datum, expecting its dissolution to naturally follow from our theoretical demonstrations, but rather starting from the recognition of dualism as an *achievement* of some importance: an achievement to which its very resilience, its stubborn endurance, testifies.

What is the character of dualism considered under the aspect of what it achieves, and thus as an expression of *value*? And if we consider dualism as an historical actuality, rather than an artefact of thought or a ‘phantom-problem’, how can we think about what this actuality contributes as an ingredient in the actualisation of the organic experience of human beings, in both health and disease?11

These questions extend and transform the scope of Deutsch’s ‘clinical-pragmatic’ agenda. Deutsch too was concerned with the differentiation of mind and body as an empirical and clinical datum, a fact of human experience, and as an achievement (of ontogenetic development). His aim was to account for the emergence of organic symptoms (partly, not exclusively) as the result of the capacity of the ‘mind’, or mental functions, to invest the ‘body’ with symbolic significance, on the assumption that ‘[t]he physiologic functions of those body parts which have become the representatives of … symbolised objects are … modified on account of the process of symbolization’.12 For Deutsch the empirical focus was clinical, limited to the mental and physiological functions of individuals, and based on hypotheses that he imagined applied universally to all human beings. Without necessarily endorsing his theoretical assumptions or his conclusions, we can agree with him on one point: the differentiation of mind and body (or mind and matter), and the baggage of connotations associated with each of these terms, is well established empirically as a *metaphor we live by*—‘we’ meaning, here, the populations of Western(ised) societies, heirs to the scientific materialism that has reigned supreme since the seventeenth century.13 To move beyond the dualism we have inherited, therefore, it is necessary to understand its epistemological limitations, and the source and character of its empirical entrenchment in our lives.

## Mind/body dualism as the sequestering of values

Modern ‘mind/body dualism’ is an instance of the broader configuration of thought and practices that issued from the scientific revolution and that by the mid-twentieth century had firmly consolidated into what C P Snow called ‘two cultures’.14 Alfred North Whitehead referred to this state of affairs as the ‘bifurcation of nature’ and traced its origins to the philosophy of the ancient Greeks, but stressed that it was only in the seventeenth century, with the systematic mathematisation of physics and particularly with the transmission theories of light and sound, that it became articulated with full consequence in the form of scientific materialism.15 In *Science and the Modern World* Whitehead poignantly conveys the ‘astounding efficiency’ of this system of thought for the organisation of scientific research and for subsequent technological development. We are the heirs of this astounding efficiency and of the transformations it wrought on the world, for better and for worse. The assumptions implicit in scientific materialism are now embedded in the organisation of our institutions and in the processes by which they mediate both social and personal life: from our education, to our healthcare, legal, and economic systems. Modern mind/body dualism is reproduced fractally across these domains and, while multiple other forms of thought coexist alongside it in the interstices of institutionally mediated practice, individuals are bound to be summoned by its normative force on a regular basis in the course of their lives, in a variety of ways.

The notion of *bifurcation* draws attention to the genealogy of modern dualism. The modern concept of nature (as bifurcated) ‘did not originate in an ontological position, either dualist or monist’, but rather in an empirically based dilemma, arising from the proliferation of ‘local operations of the qualification of (natural) bodies’.16 Mediated by technical apparatus and by mathematical abstractions, these operations consolidated the scientific understanding of physical matter as the ‘ultimate texture of nature’.17 By the same token, however, they produced the philosophical difficulty of accounting for a glaring discrepancy between the scientific description of reality (a ‘conjectured system of molecules and electrons’) and the experience of reality in perception (‘the greenness of the trees, the song of the birds, the warmth of the sun’). The modern idea of mind, from Descartes to Kant via Locke, bridges and masks the discrepancy by explaining these two orders of reality as being ‘respectively the cause and the mind’s reaction to the cause’.18 The mind, in other words, is supposedly provoked by the characteristics of matter into a reaction, but adds something of its own to produce what we experience as perceptions: hence the difference in the two orders of reality (objective and subjective), now accounted for by the ‘psychic additions’.19 John Locke famously named the physico-mathematical properties of matter ‘primary qualities’, and the psychic additions resulting in perception ‘secondary qualities’.20 In so doing he highlighted their different ontological status as, respectively, causal and epiphenomenal; of the order of substance and of the order of appearance. *Bifurcation* thus points to the process whereby, starting with an ‘immediate experience’ of nature as a single reality, that experience comes to be split into ‘two regimes of existence’.21

Why dwell on the gesture of bifurcation? Doing so allows us to appreciate the specific character of modern dualism. The bifurcation of nature produces a situation whereby our primary experience of reality in perceptual knowledge—where the qualitative richness of sensory impressions is intimately tied with evaluations of experience—is regarded as a secondary epiphenomenon, comparatively unimportant, and liable to being disqualified as a delusion when it does not correlate with knowledge mediated by physico-mathematical abstractions. The notion of bifurcation also allows us to appreciate the historical contingency of modern dualism, by highlighting its intimate relationship with the history of scientific materialism.

‘Framed by mathematicians, for the use of mathematicians’, scientific materialism yields, as we have seen, ‘on the one hand *matter* with its *simple location* in space and time, on the other hand *mind*, perceiving, suffering, reasoning, but not interfering (with matter)’.22 In contemporary medicine, this description translates into the conceptual distinction between *disease* and *illness*.23 All quality and value are expunged from the concept of nature/matter/disease and implicitly treated as projections of the mind, while nature itself is reduced to ‘a dull affair, soundless, scentless, colourless; merely the hurrying of material, endlessly, meaninglessly’.24 Whitehead is emphatic about the importance of doing full justice to the positive achievements enabled by this philosophical outlook which, as it triumphed through the efforts of eighteenth-century *philosophes*, ‘acted on the world like a bath of moral cleansing’.25 But his aim in *Science and the Modern World* is ultimately to highlight the difficulties and confusion that occur when this system of abstractions is generalised and taken at face value as a representation of concrete reality, particularly the reality of biological and human organisms. At the heart of modern thought lies what he calls a ‘radical inconsistency’, a paradox, the entertainment of which requires indeed a logical leap—one, however, that modern civilisation has been so far generally happy to fudge. The relevant passage from *Science and the Modern World* is worth quoting in full:

A scientific realism, based on mechanism, is conjoined with an unwavering belief in the world of men and of the higher animals as being composed of self-determining organisms. This radical inconsistency at the basis of modern thought accounts for much that is half-hearted and wavering in our civilisation. It would be going too far to say that it distracts thought. It enfeebles it, by reason of the inconsistency lurking in the background. After all, the men of the Middle Ages were in pursuit of a [rationalist] excellency of which we have nearly forgotten the existence. … We are content with superficial orderings from arbitrary starting points. For instance, the enterprises produced by the individualistic energy of the European peoples presupposes [sic] physical actions directed to final causes. But the science which is employed in their development is based on a philosophy which asserts that physical causation is supreme, and which disjoins the physical cause from the final end. It is not popular to dwell on the absolute contradiction here involved. It is the fact, however you gloze over it with phrases.26

Here Whitehead does a lot more than describe a logical inconsistency or contradiction. Through this description, first, he strips modern dualism of any awe-inspiring, mystical connotations: at stake in the body/mind problem there is no transcendental mystery, but there is indeed a *leap*, a logical mistake. Second he describes the tolerance of Western civilisation for this inconsistency, the fact that we are ‘content with superficial orderings’, although these enfeeble our capacity to think and can result in ‘half-hearted and wavering’ solutions. And third, he critically acknowledges attempts to ‘gloze over [the contradiction] with phrases’. These attempts testify to the fact that the contradiction nevertheless makes itself felt, such that it becomes necessary to ‘gloze over it’. Yet the absolute character of the contradiction is typically denied, and insisting on it makes one ‘unpopular’.

## So… why ‘Biopolitics’?

Writing as a philosopher, Whitehead did not make it a central task of his work to account systematically for the tolerance—and often militant upholding—of this radical inconsistency in Western civilisation.27 But historian Robert Young, in his foreword to *Science and the Modern World*, articulates the question in explicitly sociological and political terms: ‘what other values’, he asks, ‘are served by sequestering values’ from nature and from facts? 28 The routine denunciation of mind/body dualism in abstract, epistemological terms pre-empts the possibility of articulating the range of values that are concretely at stake in attempting to reconstruct our world through different concepts—and therefore what the costs, as well as the benefits, of such an attempt might be. This is one of the reasons why it is important to start with dualism as an empirical datum, rather than as a figment of the scientific or philosophical imagination.

The question about ‘what other values are served by the sequestering of values’ points—on one level—to the coalescence between the abstractions of scientific materialism and those of liberal political economy. In his historical narrative Whitehead proposes that, in parallel with the ascendancy of the modern concept of nature as governed by physical laws, economic abstractions effectively ‘naturalised’ the concept of the market and its laws as the factual, determining backdrop of human existence and social life. To cite him directly once more, in political economy ‘all thought concerned with social organisation expressed itself in terms of material things and capital. Ultimate values were excluded. They were politely bowed to, and then handed over to the clergy to be kept for Sundays’.29 From the second half of the twentieth century we have witnessed the full-blown actualisation of this reduction of *values* to (economic) *value* with the rise and triumph of Chicago-school neoliberalism as a mentality of government, or ‘governmentality’.30

Through the concepts of *biopower*, *anatomopolitics* and *biopolitics*, Foucault develops an argument that implicates the human and social sciences in the narrative framework already outlined by Whitehead. These concepts refer to a specifically modern form of governance that conceives biological life as a resource, and that is invested in fostering, administering and optimising life as such, both at the level of individual bodies and at the level of populations.31 Exercised through a range of technologies active at a capillary level throughout the social body, biopower informed the emergence of the institutions of the modern state (the family, the school, the army, the hospital and so on) and of new forms of knowledge dedicated to the understanding of human and social life as their object, including medicine and economics. These forms of power/knowledge were and are concerned with ‘the controlled insertion of bodies into the machinery of production … the adjustment of the phenomena of population to economic processes (and ultimately with) the adjustment of the accumulation of bodies to that of capital’. They act as ‘factors of segregation and hierarchization’ by articulating rationalities for distinguishing between normal, valid and valuable forms of life and those that stand to be neglected, punished or corrected.32

The eighteenth century, Foucault claims, instituted a ‘somatocracy’ that has been in crisis from the very beginning, insofar as it has involved the active neglect of important aspects of human life alongside the maximisation of those aspects that served capitalist economy and, through it, the state. In the course of the twentieth century health was progressively politicised in the context of various emancipatory movements, and in this way it became the intermediary for a degree of economic redistribution in the years following World War II. Foucault presents the decade between 1940 and 1950 as the historical threshold for the formulation of new rights, a new morality and a new economy, epitomised in the UK by the Beveridge Report of 1942. If the eighteenth century had conceived the individual in good health as existing for the benefit of the state, the mid-twentieth century reconceived the state as existing for the benefit of the individual in good health. If the nineteenth century had promoted personal hygiene as a matter of moral obligation, the post-World War II period saw the emergence of the ‘right to be ill’ and its moral exemptions, captured by Talcott Parsons through the sociological concept of the ‘sick role’.33 As Parsons also acknowledged, the medical profession came to play a crucial role as the gatekeeper to this right, whose enjoyment was conditional on medical authority being respected, and its instructions obeyed, on account of their foundation in natural science.34

Biopower under neoliberalism has taken a further inflection. It increasingly operates as and through forms of self-regulation that rely on a range of ‘technologies of the self’ while appealing to the value of individual freedom of choice.35 This context is characterised, on the one hand, by a democratisation of power relations in general, and of relations between doctors and patients (now medicine and its ‘clients’) in particular. The authority and autonomy of the medical profession have been questioned and eroded in the name of this democratisation, and a new form of impersonal authority has arisen in their place, in the form of ‘evidence’ and ‘evidence-based medicine’. What has remained relatively unchanged in these biopolitical permutations is the bifurcation of health into a factual, objective, evidence-based and supposedly value-neutral dimension, on the one hand; and an experiential, subjective dimension expressive of personal values, private moralities, individual trajectories on the other. While the importance of the subjective dimension for the purpose of defining the priorities of health policy and practice is increasingly recognised, this is often through knowledge practices that are designed to translate (soft, unreliable, idiosyncratic) ‘experience’ into (solid, reliable, generalisable) ‘evidence’, in a manner that preserves and redoubles the bifurcation. In practice the authority of evidence is not simply ‘obeyed’ but negotiated in the context of clinical interactions through a participatory, patient-centred model of care, about which I will say more below.

## … Why ‘Psychosomatics’?

Whitehead discussed the romantic poetry of Wordsworth and Shelley as ‘a protest on behalf of the organic view of nature, and also a protest against the exclusion of value from the essence of matter of fact’.36 Since the days of Romanticism, the dehumanising character, limitations and even iatrogenic consequences of a biomedical model resting on the assumptions of mechanistic science have been articulated in a variety of critical discourses, some external and some internal to medicine itself. There is no room here to offer even a synthetic summary of these movements and the ideas that animated them.37 Suffice it to say that protests against biomedical reductionism have been numerous and heterogeneous. The emergence of a ‘new subjective medicine’ and of medical humanities/narrative medicine constitute two of the most recent developments in this regard.38 The question that arises here concerns how far such protests go in subverting the underlying abstractions of scientific materialism, and the radical inconsistency they produce; or, conversely, to what extent they may be said to constitute ways of ‘glozing over’ the contradiction, to use Whitehead’s expression. This is not a question that can be answered in general or in the abstract, with reference to the professed intentions and allegiances of such movements, for in practice they can be internally heterogeneous.

The field known as ‘psychosomatic medicine’ has also been internally very heterogeneous in the course of the hundred or so years of its history. But I venture that it is in this field that we find some of the most explicit and radical formulations of the ‘protest on behalf of value’ with specific relevance to medicine. Some of the propositions yielded by this protest are likely to sound distinctly unpopular, even outrageous, today—not, as one might imagine, because they are pseudoscientific, but because they spell out the misplaced concreteness of medical assumptions derived from scientific materialism. If I repropose them here it is not because I think they should be uncritically endorsed, but again as lures for thought and spurs for discussion.

*Proposition 1:* Whitehead expected the demise of the seventeenth-century philosophy of nature to issue from developments in the life sciences, particularly physiology and psychology. But he also stressed that ‘the progress of biology and psychology has … been checked by the uncritical assumption of half-truths’ from the physical sciences.39 We find a strong echo of this reflection in the address that Viktor von Weizsäcker delivered in 1949 to the German Society for Internal Medicine, where he offered a critical appraisal of the prospects of psychosomatic medicine, seven full years before Deutsch convened his workshop. The future of psychosomatic medicine looked bright at that point, and today many consider the decades around mid-century to have been its heyday. In his talk Weizsäcker, who had a background in experimental neurophysiology, questioned whether the field was on the right trajectory to fulfil its ambitions. Much of what was taking place in the name of psychosomatic medicine, he claimed, was no more than psychophysiology: an effort to establish objective correlations between certain psychological events—like a fright or a pleasure—and certain somatic events, such as a vasomotor reaction. But the nature of the questions being asked by researchers remained the same: what factors are associated with this or that disease? The experiments of psychophysiology would complicate the multifactorial picture for sure, but they would not challenge the fundamental mindset and orientation of modern medicine.

The true potential of psychosomatic medicine, Weizsäcker ventured, lay somewhere else entirely. The new medicine would have to stop considering organic disease/illness purely as an objective event—in the sense of an event that occurs in and as an object—and approach it instead in terms of questions *that can only be asked of a subject*, namely questions concerning motives, values and aims. His own early neurophysiological experiments on the relation between perception and movement had demonstrated how ‘vital crises’ and organic symptoms could be precipitated by a subject’s ‘proleptic’ efforts to maintain coherence by anticipating future movement.40 Later he wrote of the ‘proleptic structure of biography’, and claimed that the study of the role of anticipation or prediction in pathology— that is, the study of the efficacy of the future—ought to have ‘polemical priority’, because it had entirely been neglected for the previous 300 years.41 He proposed the ‘pathic pentagram’ based on the German modal verbs (*wollen*, *dürfen, müssen*, *sollen*, *können*) as a conceptual device through which clinicians could explore the event of illness/disease in terms the dynamic relationships between ‘wanting to do’, ‘being allowed to do’, ‘being forced to do’, ‘being obliged to do’ and ‘being able to do’. Each of these categories implies futurity and a mode of being that, in Whiteheadian terms, entirely escapes the abstractions and language of ‘simple location’.42 In sum, biological facts—and not merely their interpretations in knowledge or self-reports— ought to be understood as normative *acts* involving *values* and *aims*. The interrogation of values and aims therefore ought to be at the centre of the psychosomatic agenda.

*Proposition 2:* a second proposition that we find in the history of psychosomatic medicine has its origins in psychoanalysis, and links the organic and personal dimension of value to the social and biopolitical dimension. This is the idea that illness has value as the *compromise solution to a conflict*. In most interpretations of this idea the conflict is assumed to be entirely infrapsychic, and thus an individual affair. But we need not be limited to this interpretation. Consider, once again, Weizsäcker:

If organic disease is the substitute for an unresolved conflict; if if can be defined as a flight from conflict into disease; if it is, therefore, the materialisation of a conflict; then, even with its spiritualisation, the conflict remains unresolved. In other words the successful outcome of the psychotherapy of an organic disease is at the same time the reproduction of a conflict. When this conflict leads to thoughts previously unheard of, and to even more outrageous actions, this produces a surrounding environment that may not find the change acceptable at all. Whether the change in question is a divorce, a political subversion, or a religious one, in all these cases the person who *thus* recovers comes to contradict the customary order of things, and his therapist comes to be blamed by his friends and by the beneficiaries of the prior social situation.43

This proposition will not be especially surprising to anyone who is familiar with systems-theoretical (or cybernetic) approaches to psychotherapy and more generally to mental health, including Batesonian (anti)psychiatry.44 These approaches involve an ‘ecological’ understanding of mental illness that addresses how an illness may benefit the equilibrium of a particular configuration of relations; how, in other words, it can perform a social function and represent a value for that system, if not for the individuals directly concerned. Psychosomatic medicine, at least in certain versions of it, invites us to analyse somatic disease in similar ways. In this sense, it points to the need for a *critical political economy of disease/illness—*one that might articulate what investments individuals make in the performance of normative forms of subjectivity, and what *value* (rather than what cost) certain occurrences of disease and illness represent for the preservation of a certain sociopolitical order. The success or failure of psychosomatic medicine might then be (re)assessed from this perspective, as Weizsäcker already proposed in 1949:

What has clinical psychosomatic medicine accomplished up to now? Whoever frames the question in these terms, and by ‘accomplishment’ refers to nothing but practical utilisation within a modern industrial nation, would have to answer more or less as follows: for the majority of internal diseases it has accomplished nothing at all, for some of them very little, and even then only for isolated cases … I too would make a similar judgement if I felt subjected to the business ideal of the entrepreneurial state. … I believe then that we must make a clarification. The evaluation of theoretical and practical *successes* ultimately depends on the following alternative: whether medicine and doctors subscribe to the value judgement proper to the entrepreneurial state, or whether they locate the value of human life elsewhere.45

*Proposition 3* *:* the last proposition from psychosomatic medicine that I will offer here as a lure for thought follows from the last two, and it concerns the distinction, and the relationship, between organic disease and mental illness. If we posit an organism that is expressive of evaluations and sensitive to sociocultural values as part of its living milieu, then the different value connotations and social implications of ‘organic disease’ and ‘mental illness’ will be significant as an ingredient of experience, informing what constitute more or less costly forms of adaptation within a given environment. Following from this premise, the development of an organic disease could, in principle, be understood to constitute a form of ‘psychic saving’ with respect to other possibilities involving significant psychosocial costs to the person. This proposition extends and transforms a similar one made by social psychologist Karola Brede, according to whom the task of psychosomatic medicine would be to

interpret, from a psychological perspective, the fact that psychosomatic patients appear inconspicuous from a psychopathological point of view, and that they share this inconspicuousness with organic patients. [Psychosomatic medicine] must, in other words, interpret this as an intentional psychic saving and provide it with a sociological foundation by referring it to the social control of norm-transgressive behaviour.46

Brede limits this task to the analysis of ‘psychosomatic illness’ as a subgroup of conditions—but this is a limitation that I invite us to query and debate. Theoretically, what this proposition allows us to grasp is that at the core of what biomedicine cannot see lies the fact that its ‘object’ knows what it can see, and adjusts its aims accordingly.47

## … *Why ‘Participation’ and ‘Participating Bodies’?*

*Patient involvement*, *participation* and *empowerment* are keywords at the forefront of the politics of contemporary healthcare. While the precise scope and meaning of these concepts can be a matter of debate, they now inform a wide range of activities and associated technologies at different levels, from individual doctor-patient consultations to the development of clinical guidelines and research programmes. The multiplicity and diversity of this development caution against speaking about it in any too general or abstract terms, but again I will venture here some propositions that aim at facilitating a discussion of participation as a ‘psychosomatic problem’.

Discourses of participatory medicine envisage a future where ‘networked patients shift from being mere passengers to responsible drivers of their health’, and involve a double emancipatory promise.48 On the one hand, there is the promise of a democratisation of power relations in the clinical encounter and beyond. Explicit and revealing in this respect is the title of the second annual conference of the Society for Participatory Medicine, held in 2018: *Democratising Healthcare! Me. You. Us. Healthocracy*. The title encapsulates the contemporary inflection of biopower by inviting doctors and patients to collaborate as equals—their respective roles rendered simply as ‘me’ and ‘you’—under the ‘rule of health’ (healthocracy).49 In this new configuration, the traditional authority of the doctor is counterbalanced by that of the patient; both, however, must submit to the impersonal and objective authority of clinical evidence.50 In this sense, the promise of democratisation reproduces the bifurcation of nature by instituting 'evidence' as a supposedly neutral arbiter amongst potentially conflicting, situated expressions of value. On the other hand, the promise of participatory medicine concerns the possibility of addressing the clinical shortcomings of a reductive biomedical model by incorporating the ‘patient’s point of view’—an expression that can refer to a variety of more specific concepts ranging from *choice* and *preferences* to *experience* and *narratives*. While ostensibly the reference to these concepts involves an elicitation of *value(s)*, this elicitation is typically subject to three important constraints. First, it is limited to the values explicitly and consciously articulated by participants, on the assumption that the totality of their experience is available to them for expression—a fact that has not escaped critical attention.51 Second, the way in which experience and value come to matter institutionally depends, in most instances, on processes of aggregation and abstraction that translate them into ‘evidence’, with only limited reflection on what is lost in that translation. And third, the dimension of value is not deemed relevant to the explanation of organic events, on the assumption that the abstractions of scientific materialism remain adequate for that purpose. 50

The discourse of participatory medicine thus reproduces at various levels the assumptions of modern dualism, whereby the capacities of the ‘mind’ - associated with the articulation of subjective preferences, values, experiences - are treated as ontologically different and unrelated to those of the ‘body’. We also see the radical inconsistency whereby, despite this bifurcation, the possibility of an interaction between mind and body is posited through the notion of personal responsibility for health. This inconsistency typically yields a simple alternative between regarding health outcomes as the result of individual choices on the part of free agents, or conversely regarding them as the result of blind physical determinism. Public debate, particularly on so-called ‘lifestyle diseases’, indeed appears to be permanently stuck in an oscillation between these two poles.

Earlier in this paper, in my discussion of biopolitics, I already indicated what other values this sequestering of value might serve: following Whitehead and Foucault, I proposed that we should look at the function of medical dualism in relation to the requirements of liberal political economy and the modern state. Following Weizsäcker, I also suggested the importance of articulating a political economy of disease/illness based on an analysis of the investments individuals make in the performance of normative forms of subjectivity, and the consequences of those investments for their health. This appears all the more urgent and important now, in relation to the context of neoliberalism and of participatory medicine as an expression of it. In this context, a rhetoric of work in the name individual fulfilment and self-realisation has replaced an older vocabulary of class antagonism that made the social and personal costs of labour relations more readily apparent. More generally, the neoliberal context is one where the possibility of resistance and refusal is subverted by the fact that, as Nikolas Rose famously put it, we are ‘obliged to be free’.52 A transdisciplinary psychosomatics has a concrete role to play in articulating the limits and costs—both personal and collective—of this ‘freedom’.

Against this background, the idea of *participating bodies* (or participation *all the way down*) is one that I propose in direct contrast to the assumptions and value commitments of participatory medicine. Reclaiming a fundamental insight from the history of psychosomatics, participation *all the way down* points to the possibility of conceiving bodies themselves—and bodily events such as disease/illness—as expressing values and perhaps even socially meaningful ‘preferences’.

## Conclusion

In this paper I have articulated the problematic of psychosomatics through a number of propositions that reconnect it to the present and to contemporary concerns. In contrast to classic approaches to ‘psychosomatic problems’, which typically set out by denouncing the conceptual inadequacy of mind/body dualism, I proposed to begin by focusing on the *resilience* of dualism as an empirical datum deserving closer analysis. Drawing on the philosophical and historical accounts of A N Whitehead and Michel Foucault, I argued that modern dualism is an epistemic configuration that involves a ‘sequestering of values’ from nature and from facts; and that this configuration has itself a functional utility (or value) in the context of liberal political economy and biopolitical forms of governance. This analysis placed the question of *value* at the centre of the modern problematic of psychosomatics, and made it possible to reclaim the relevance of specific propositions from the history of psychosomatic medicine that articulated the question of value specifically in relation to disease/illness. On this basis I argued for the importance of developing a critical political economy of disease/illness.

The grip of modern dualism on the organisation of contemporary societies can appear totalising, and the optimism of the psychosomatic movement in its heyday now seems decidedly naïve. In conclusion, one further lesson we might draw from the history of psychosomatic medicine is that the transformation of this mentality is unlikely to occur by way of a frontal confrontation, or by the proclamation of a medicine-wide project of reform. In order to articulate possibilities and strategies for change we must look, instead, in the interstices, the cracks and the ‘frustrations of established order’: in other words, to those places, those phenomena, where dualism fails—not epistemologically but pragmatically—and where other forms of thought and practice are prompted into existence.53 Where are these cracks, and how can we sustain and nurture the life that lurks within them? It is by finding them, and by articulating their potential, that a new relationship between medicine and the humanities may be cultivated today.

## Notes

1. Isabelle Stengers and Vinciane Despret (2014), *Women Who Make a Fuss – The Unfaithful Daughters of Virginia Woolf* (Minneapolis: Univocal), 43.

2. Freud’s phrase was the ‘mysterious leap from the psychic to the physical’, see Sigmund Freud (1920), *A General Introduction to Psychoanalysis*, trans. Stanley Hall (New York: Boni & Liveright). The adjective in Freud’s original phrase (*rätselhafter*), translated by Hall as *mysterious*, can also be translated as *puzzling*, a term with rather different connotations (a puzzle can be solved in principle, a mystery is inherently beyond understanding). The noun from which the adjective derives (*Rätsel*) can be translated as *riddle, puzzle* or *brainteaser* as well as *mystery*. Himself a native German speaker, Deutsch cited Hall’s translation of this term without further qualification. In his translation of the same passage, James Strachey uses the expression ‘puzzling leap’ – see Sigmund Freud (1974), *Introductory Lectures on Psychoanalysis*, trans. James Strachey (Harmondsworth: Penguin Books), 297. See also the discussion of this point in Lilian R Furst (2003), *Idioms of Distress: Psychosomatic Disorders in Medical and Imaginative Literature* (Albany: State University of New York Press), chapter 3.

3. Stanley Cobb et al. (1959), “Is the Term ‘Mysterious Leap’ Warranted?,” in *On the Mysterious Leap from the Mind to the Body – A Study On the Theory of Conversion* ed. Felix Deutsch (New York: International Universities Press), 11.

4. Stanley Cobb et al. (1959) **, “Is the Term ‘Mysterious Leap’ Warranted?,” 11, 16.

5. Cobb et al., 18.

6. Cobb et al., 25.

7. Monica Greco (1998), Illness As a Work of Thought (London: Routledge).

8. Laurence Foss (2002), *The End of Modern Medicine – Biomedical Science Under a Microscope* (Albany, NY: SUNY Press).

9. Nissim Mizrachi (2001), “From Causation to Correlation: The Story of *Psychosomatic Medicine*, 1939–1979,*” Culture, Medicine and Psychiatry* 25, no 3: 317–343.

10. Nissim Mizrachi (2001), “From Causation to Correlation: The Story of *Psychosomatic Medicine*, 1939–1979,*”* 317. For a pertinent critique of the limitations of Mitzrachi’s account, which is broadly in line with the approach adopted in this paper, see Gary S. Belkin, “Causation About What? Relevant to Whom? Linking Psyche and Society – A Commentary on Nassim Mitzrachi’s ‘From Causation to Correlation’,” *Culture, Medicine and Psychiatry* 25: 341–349.

11. The physicist Max Planck characterised the mind-body problem as a ‘phantom-problem’ in his *Scientific Autobiography* (cited in Deutsch, “The Riddle of the Mind-Body Correlations”, 8).

12. Felix Deutsch (1959), “Symbolization As a Formative Stage of the Conversion Process”, in *On the Mysterious Leap From the Mind to the Body*, ed. F. Deutsch (New York: International Universities Press), 76. Symbolisation is at the heart of the concept of ‘conversion’, which refers to the transformation of psychic phenomena into organic ones. Deutsch’s version of this concept drew on Freud’s but differed significantly from it, in that for Deutsch the conversion process was not inherently tied (or limited) to the formation of pathological symptoms. He conceived conversion as a normal psychophysiological process, a ‘perpetual life stream’ or ‘a continually active process normally innate in the organism … [that] plays a part in any normal and neurotic condition’. Likewise he described the embodiment of symbols as ‘the “soul” of the organism [without which] psychic life expires’ (Felix Deutsch, “Closing Remarks” in *On The Mysterious Leap From the Mind to the Body*, ed. F. Deutsch, 252–3). This proposition can be fruitfully related to more recent transdisciplinary research on the role of metaphor in mediating immune function, which includes anthropological critiques of culturally specific assumptions that stem from the legacy of psychoanalysis. See James M Wilce and Laurie J Price (2003), “Metaphors Our Bodyminds Live By”, in *Social and Cultural Lives of Immune Systems*, ed. James M. Wilce Jr. (London: Routledge), 50–81.

13. On ‘metaphors we live by’ see George Lakoff and Mark Johnson (1980), *Metaphors We Live By* (Chicago: University of Chicago Press); for an extension of Lakoff and Johnson’s original concept and critical discussion of its relevance for the understanding of psychosomatic processes see Wilce and Price, “Metaphors Our Bodyminds Live By”.

14. Charles P Snow (1959), *The Two Cultures* (Cambridge: Cambridge University Press).

15. Alfred North Whitehead (1920), *The Concept of Nature* (New York: Prometheus Books, 2004 [1920]).

16. Didier Debaise (2017), *Nature As Event – The Lure of the Possible* (Durham and London: Duke University Press), 13, emphasis removed.

17. Alfred North Whitehead (1920), *The Concept of Nature*, 20.

18. Whitehead, 31.

19. Whitehead, 29–30.

20. John Locke, *An Essay Concerning Human Understanding* (London: Penguin Books, 1997 [1690]).

21. Didier Debaise (2017), *Nature As Event*, p. 12.

22. Alfred North Whitehead (1926), *Science and the Modern World,* 70.

23. See Cecil G Helman (1981), “Disease vs Illness in General Practice,” *Journal of the Royal College of General Practitioners* 31: 548–552 for an early review of the conceptual distinction between *disease* and *illness* that conveys more nuances for each of these concepts than have been preserved in their mainstream current usage.

24. Whitehead, *Science and the Modern World*, 69.

25. Whitehead, 74.

26. Whitehead, 94–95.

27. Whitehead's most sustained engagement with this question can be found in Adventures of Ideas (1933) where his focus, however, is not specifically on health and illness. For a discussion see Paul Stenner (2013), "Human Rights between Brute Fact and Articulated Aspiration," in *Making Human Rights Intelligible* ed. Mikael Rask Madsen and Gert Verschraegen (Oxford and Portland, Oregon: Hart Publishing).

28. Robert Young (1985), “Foreword,” in Whitehead, *Science and the Modern World,* (London: Free Association Books), xvi.

29. Whitehead, *Science and the Modern World*, 252.

30. For an excellent introduction to the concept of govermentality and to Foucault’s analysis of neoliberalism see Thomas Lemke (2001), “‘The Birth of Biopolitics’: Foucault’s Lecture at the Collège de France On Neo-Liberal Governmentality,” *Economy and Society* 30, no. 2: 190–207.

31. In Foucault these three terms refer to different aspects of the same phenomenon. For the purposes of my title I have elided them into *biopolitics*.

32. Michel Foucault (1978), *The History of Sexuality Volume 1: An Introduction* (New York: Pantheon Books), 141.

33. Michel Foucault (2004), “The Crisis of Medicine Or the Crisis of Antimedicine?,” *Foucault Studies* 1: 5–19; Talcott Parsons (1951), *The Social System* (London: Routledge and Kegan Paul Ltd.).

34. Isabelle Stengers (2003). “The doctor and the Charlatan.” Cultural Studies Review 9.

35. Scholars in the tradition of ‘govermentality studies’ have extensively documented this development and its biopolitical dimensions (see Lemke, “The Birth of Biopolitics”, for a review and discussion). For a critique of the ‘logic of choice’ in relation to health and healthcare settings, with an empirical focus on the case of type 2 diabetes, see Annemarie Mol (2006), *The Logic of Care – Health and the Problem of Patient Choice* (London: Routledge).

36. Whitehead, *Science and the Modern World*, 117–118.

37. For a collection of essays discussing different historical instances of ‘holism in biomedicine’ between 1920 and 1950 see Christopher Lawrence and George Weisz (1998), *Greater Than the Parts: Holism in Biomedicine, 1920–* *1950* (Oxford: Oxford University Press).

38. The ‘new subjective medicine’ refers broadly to the range of knowledge practices that seek to produce patient-centred outcomes by bringing ‘the patient’s point of view back into the centre of clinical medicine’, see Mark Sullivan (2003), “The New Subjective Medicine: Taking the Patient’s Point of View on Healthcare and Health,” *Social Science and Medicine* 56: 1595–1604. For a discussion of medical humanities and narrative medicine in relation to psychosomatics see Monica Greco (2008), “On the Art of Life: A Vitalist Reading of Medical Humanities,” *Sociological Review* 56: 23–45; and Monica Greco (2013), “Logics of Interdisciplinarity: The Case of Medical Humanities”, in *Interdisciplinarity: Reconfigurations of the Social and Natural Sciences* ed. Georgina Born and Andrew Barry (London and New York: Routledge), 226–246.

39. Whitehead, *Science and the Modern World*, 21. The theme of a ‘reenchantment’ driven by the life sciences in the context of Weimar culture is the object of a study by Anne Harrington. See Anne Harrington (1996), *Reenchanted Science: Holism in German Culture from Wilhelm II to Hitler* (Princeton: Princeton University Press).

40. Victor. von Weizsäcker (1950), “Der Gestaltkreis. Theorie der Einheit von Wahrnehmen und Bewegen,” in *Der Gestaltkreis. Theorie der Einheit von Wahrnehmen und Bewegen – Gesammelte Schriften*, vol. 4, ed. Dieter Janz, Wilhelm Rimpau and Walter Schindler (Frankfurt-am-Main: Suhrkamp Verlag, 1997), 83–338. The Merriam Webster dictionary defines *prolepsis* as ‘anticipation’, or ‘the representation or assumption of a future act or development as if presently existing or accomplished’.

41. Viktor von Weizsäcker (1956), *Pathosophie – Gesammelte Schriften*, vol. 10, ed. Walter Schindler, Dieter Janz and Peter Achilles (Frankfurt-am-Main: Suhrkamp Verlag, 2005), 287. The ‘polemical priority’ Weizäcker attributes to the interrogation of aims and values points to his sense of an urgent need to counter the dominance of the abstractions of scientific materialism, and contextualises what some might regard as an unduly unilateral emphasis on the subjective dimension of pathology. Georg Groddeck (1934) shared the feeling that the comparative neglect of the ‘inward cause’, as he called it, justified his own exaggerations in articulating it: ‘In medicine the external cause has received so much attention – it is in some ways, of course, much the simpler to deal with or at least to name – that there can be no great harm if a few doctors here and there seem to exaggerate the importance of the neglected inward cause, and maintain as I do that man creates his own illnesses for definite purposes …’ Georg Groddeck, *The World of Man* (London: Vision Press, 1951), 81.

42. Weizsäcker referred to this mode of being as *pathic* (as distinct from *ontic*) and *antilogical*. These concepts he considered specifically pertinent to phenomena of life, insofar as a living being never simply *is* but rather experiences life in and as the contradiction between the possibilities of being and non-being. See Victor. von Weizsäcker (1946), “Anonyma”, *Gesammelte Schriften*, vol. 7, ed. Peter Achilles (Frankfurt-am-Main: Suhrkamp Verlag, 1987), 41–91.

43. Viktor von Weizsäcker (1949), “Psychosomatische Medizin,” *Gesammelte Schriften*, vol. 6, ed. Peter Achilles (Frankfurt-am-Main: Suhrkamp Verlag, 1986). 461.

44. Some of the key texts in this tradition are Gregory Bateson (2000), *Steps to an Ecology of Mind: Collected Essays in Anthropology, Psychiatry, Evolution, and Epistemology* (Chicago: University of Chicago Press, 2000); Paul Watzlawick et al. (1967), *Pragmatics of Human Communication: A Study of Interactional Patterns, Pathologies, and Paradoxes* (New York and London: W. W. Norton & Co., 1967); Ronald D Laing (2010), *The Divided Self: An Existential Study in Sanity and Madness* (Harmondsworth: Penguin, 2010).

45. Viktor von Weizsäcker (1949), “Psychosomatische Medizin”, 457–8.

46. Karola Brede, 1972 (65).

47. See M Savransky, “When Bodies Think: Panpsychism, Pluralism, Biopolitics,” (this issue) for an elaboration on how biological organisms, and not just ‘minds’, may be said to participate in processes of thinking and discerning.

48. Society for Participatory Medicine, “About Us,” Society for Participatory Medicine, accessed September 2, 2018, http://participatorymedicine.org/about.

49. Foucault taught us that if we need to be suspicious of biopower as a form of governance, it is not on account of its association with a particular kind of agency, whether medicine or the state, but because it defines and limits the horizon of our thinking when it comes to articulating values and freedoms. In 1974, addressing an audience on the topic of ‘anti-medicine’, he claimed that ‘there (was) no longer anything outside medicine’; antimedical critiques themselves only ever spoke in the name of a better medicine. Today we might rephrase this insight to say that ‘there is no longer anything outside health’: if the role of medicine now appears limited, it is only on account of a more far-reaching and encompassing understanding of the factors involved in producing health, which requires the enrolment of citizens and their lifeworld into the biopolitical project. See Michel Foucault (2004), “The Crisis of Medicine or the Crisis of Anti-Medicine,” 14; and Monica Greco (2009) “Thinking Beyond Polemics: Approaching the Health Society Through Foucault.” *Österreiche Zeitung für Soziologie* 34, no. 2: 17.

50. Carl May et al. (2006), Tim Rapley, Tiago Moreira, Tracy Finch and Ben Heaven, “Technogovernance: Evidence, Subjectivity, and the Clinical Encounter in Primary Care Medicine,” *Social Science and Medicine* 62, no. 4 (February 2006): 1022–1030.

51. Cf. Alfred North Whitehead, *Modes of Thought* (New York: The Free press, 1966), 89: ‘We experience more than we can analyse. For we experience the universe, and we analyse in our consciousness a minute selection of its details.’ For just two examples of work that critically examine the assumptions involved in the references to patient ‘experience’ and patient ‘perspective’, see David Allen and Kristin Cloyes (2005), “The Language of ‘Experience’ in Nursing Research,” *Nursing Inquiry* 12, no. 2: 98–105; and Jeannette Pols (2005), “Enacting Appreciatinos: Beyond the Patient Perspective,” *Healthcare Analysis*, 13, no. 3: 203–221.

52. Nikolas Rose (1990), *Governing the Soul: The Shaping of the Private Self* (London: Routledge).

53. Alfred North Whitehead (1938), *Modes of Thought*, 87.
